# Structural divergence and loss of phosphoinositide-specific phospholipase C signaling components during the evolution of the green plant lineage: implications from structural characteristics of algal components

**DOI:** 10.3389/fpls.2014.00380

**Published:** 2014-08-05

**Authors:** Koji Mikami

**Affiliations:** Division of Marine Life Science, Genetics and Genomics, Faculty of Fisheries Sciences, Hokkaido UniversityHakodate, Japan

**Keywords:** alga, phosphoinositide-specific phospholipase C, phosphatidylinositol phosphate-kinase, protein domain, genome

Phosphatidylinositol (PtdIns) is involved not only in the structural composition of eukaryotic cellular membranes but also in the regulation of a wide variety of physiological processes influencing growth and development (Xue et al., 2009; Janda et al., 2013). Because molecules that participate in PtdIns signaling are generated *via* PtdIns metabolism, extensive attention has been paid to genes encoding enzymes involved in this metabolism in order to elucidate developmental and stress-response mechanisms. PtdIns is synthesized from CDP-diacylglycerol and cytoplasmic inositol by PtdIns synthase (PIS) and is phosphorylated sequentially on its inositol ring to produce PtdIns4*P* and PtdIns(4,5)*P*_2_ by PtdIns 4-kinase (PI4K) and PtdIns phosphate-kinase (PIPK), respectively (Xue et al., 2009; Janda et al., 2013). Subsequently, PtdIns(4,5)*P*_2_ is cleaved by phosphoinositide-specific phospholipase C (PI-PLC) into the second messengers diacylglycerol and inositol-1,4,5-trisphosphate [Ins(1,4,5)*P*3, IP_3_] (Xue et al., 2009; Janda et al., 2013), which then activate protein kinase C (PKC) and the IP_3_ receptor involved in release of Ca^2+^ from the ER into the cytoplasm, respectively, in animal cells (Rebecchi and Pentyala, 2000; Suh et al., 2008). In fact, genes encoding orthologs of PKC and the IP_3_ receptor are not found in terrestrial plant genomes, suggesting differences in second messenger systems between animals and plants.

Significant genomic information has been accumulated for unicellular algae including the green alga *Chlamydomonas reinhardtii* (Merchant et al., 2007), the red alga *Cyanidioschyzon merolae* (Matsuzaki et al., 2004), for the diatoms *Thalassiosira pseudonana* and *Phaeodactylum tricornutum* (Armbrust et al., 2004; Bowler et al., 2008), and also for multicellular seaweeds such as the red seaweeds *Pyropia yezoensis* (Nakamura et al., 2013) and *Chondrus crispus* (Collén et al., 2013), and brown seaweed *Ectocarpus siliculosus* (Cock et al., 2010). In addition, large-scale EST information has been accumulated for the red seaweeds *Porphyra umbilicalis* and *Porphyra purpurea* (Chan et al., 2012; Stiller et al., 2012). Because understanding the evolutionary aspects of PtdIns signaling can help us to understand the process of establishment of the plant PtdIns signaling system, algal PI-PLC and PIPK protein structures have been compared with those of terrestrial plants.

## PI-PLC

**Figure 1:**
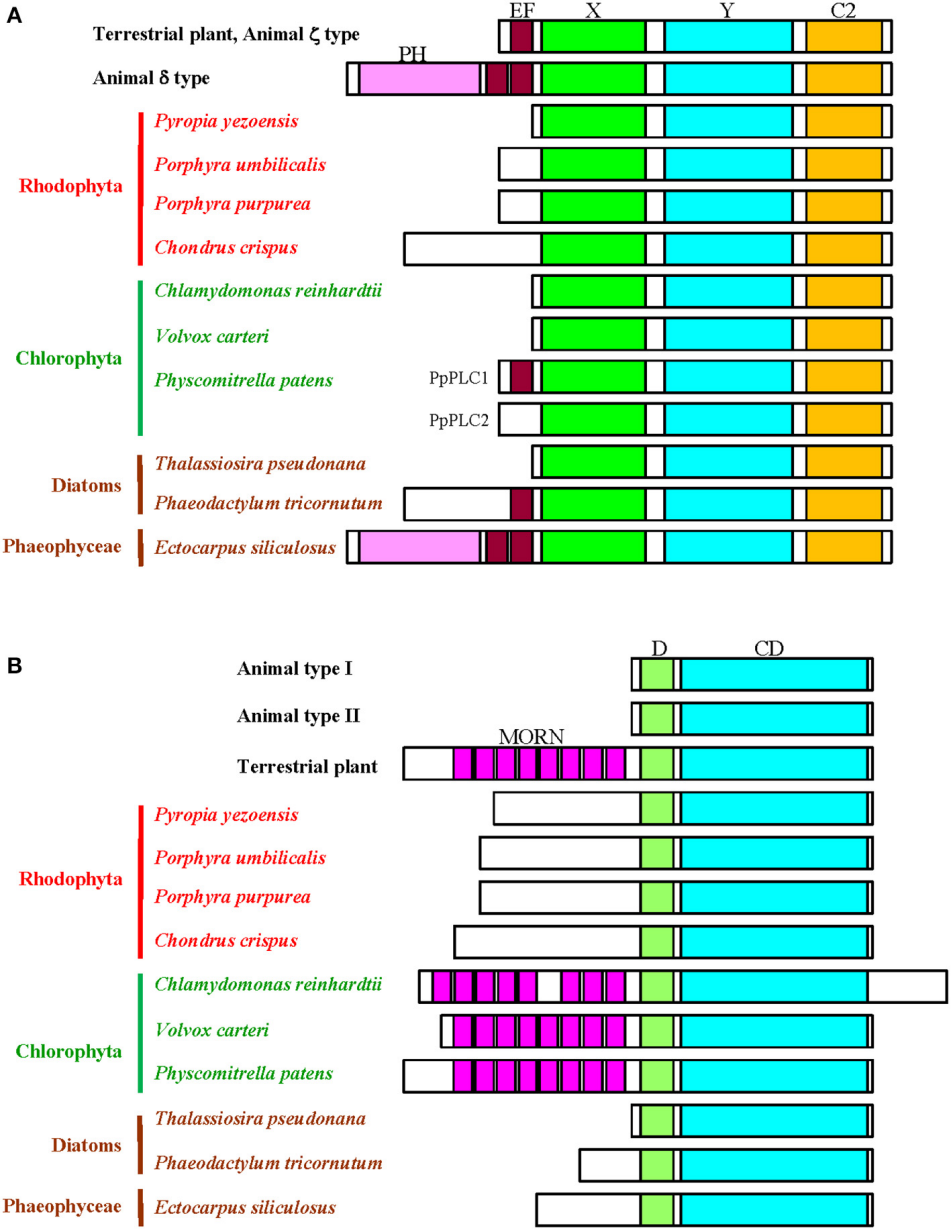
**Schematic comparison of the domain structures of the PI-PLC signaling components**. **(A)**, PI-PLC; **(B)**, PIPK. D, dimerization domain; CD, catalytic domain.

The first record of the PI-PLC gene in plants was presented by Hirayama et al. (1995). Since then, information on PI-PLC genes from terrestrial plants and green algae has accumulated from results of molecular cloning and *in silico* analysis based on genome sequence data. In mammals, PI-PLC isozymes have been divided into six groups according to their domain structural characteristics (Rebecchi and Pentyala, 2000; Suh et al., 2008). For example, PLCδ consists the Pleckstrin homology (PH) domain, the EF hand, and X, Y, and C2 domains (Figure 1), although PLCγ and PLCε contain the SH2 and SH3 domains and the Ras-GEF and RA domains, respectively, in addition to domains found in PLCδ. The PH domain participates in membrane binding, whereas the X and Y domains are responsible for catalytic activity. Plant PI-PLCs reported so far, however, lack the PH domain, similar in that to the mammalian PI-PLCζ isoform (Pokotylo et al., 2014). They consist of an N-terminal EF-hand, a central X/Y catalytic domain, and a C-terminal C2 domain (Figure 1). The EF hand plays an important role in Ca^2+^-dependent activation of PI-PLCζ, suggesting a similar function in plant PI-PLCs. Indeed, it has been demonstrated that the activities of PI-PLCs from plants were stimulated by Ca^2+^ in a concentration dependent manner (for instance, Hirayama et al., 1995; Otterhag et al., 2001; Hunt et al., 2004; Mikami et al., 2004). Although the EF hand in mammalian PI-PLC has four repeats of helix-loop-helix structure, plant enzymes carry only two of these repeats due to truncation (Figure 1). The C2 domain in plant PI-PLCs was suggested to have evolved separately from animal PI-PLCs and is possibly involved in membrane localization (Rupwate and Rajasekharan, 2012).

Genome analysis has identified nine copies of the *PI-PLC* genes (AtPLC1-AtPLC9) in *A. thaliana*, although AtPLC8 and AtPLC9 with amino acid substitutions in the Y domain do not group into the clade containing other PLCs during phylogenetic analysis (Mueller-Roeber and Pical, 2002; Hunt et al., 2004). However, only one and two copies of PI-PLC genes have been found in *C. reinhardtii* and *Physcomitrella patens*, respectively (Mikami et al., 2004; Awasthi et al., 2012). Moreover, because red and brown seaweeds as well as green microalgae have only a single copy of the PI-PLC gene, amplification and functional diversification of these genes likely occurred during evolution of streptophytes (land plants and charophytic algae) with establishment of multicellularity and vascular systems after the colonization of land.

It is worth noting that *P. patens* has a novel PI-PLC without PtdIns(4,5)*P*_2_-hydrolyzing activity, with an insertion at its N-terminal EF hand (Mikami et al., 2004). Figure 1 shows the absence of the EF hand in unicellular and multicellular algae from aqueous environments, except for the diatom *P. tricornutum* and the brown seaweed *E. siliculosus* (Figure 1). The EF hand might have appeared after the colonization of land by green algal lineages and, subsequently, PI-PLCs lacking the EF hand disappeared during the evolution of terrestrial green plants. Thus, the EF-hand-mediated regulatory mode of PI-PLC activation seems to have evolved in the green plant lineages after the colonization of land.

The evolutionary establishment of the domain structure of PI-PLC in brown seaweeds is highly complex. As shown in Figure 1, *P. tricornutum* and *E. siliculosus* have the EF hand motif. Thus, the EF hand was acquired during the evolution of diatoms and brown seaweeds. Surprisingly, PI-PLC in *E. siliculosus* contains the non-truncated EF hand and the PH domain, just as does the mammalian PI-PLCδ isoform (Figure 1). The reason that only the brown seaweed has an isoform of the PI-PLCδ type remains to be resolved.

## PIPK

The first molecular characterization of the plant PIPK gene was performed by Mikami et al. (1998), following molecular cloning and *in silico* identification based on the genome sequences of land plant PIPKs. Due to their substrate specificity in animals, PIKs can be divided into three subfamilies, known as types I, II, and III. Types I and II enzymes convert PtdIns(4)*P* and PtdIns(5)*P*, respectively, to produce PtdIns(4,5)*P2* (Anderson et al., 1999), whereas type III enzymes use PI(3)*P* to produce PI(3,5)*P2* similar to yeast Fab1 (Mueller-Roeber and Pical, 2002). Despite such differences in substrate specificity, the structures of type I and II PIPKs are highly similar in animals; however, based on substrate specificity and structure, the plant enzymes are classified as type I/II PIPKs (Mueller-Roeber and Pical, 2002; Thole and Nielsen, 2008; Saavedra et al., 2012).

The genome of *A. thaliana* contains 11 PIPKs, subdivided into A and B subfamilies according to their structural differences. The domain structure of subfamily A (AtPIPK10 and AtPIPK11) shows similarity to animal PIPKs, whereas the domain structure of subfamily B members (AtPIP5K1-AtPIP5K9) includes a long N-terminal extension containing a repeat of the membrane occupation and recognition nexus (MORN) motif (Mueller-Roeber and Pical, 2002; Saavedra et al., 2012) Although the domain containing MORN motifs has been hypothesized to be a module involved in plasma membrane-localization and phosphatidic acid (PA)-dependent enzymatic activation (Im et al., 2007), it was recently demonstrated that the activation loop conserved in the lipid kinase domain is responsible for both plasma membrane localization and PA-dependent activation of *A. thaliana* and *P. patens* PIPKs (Mikami et al., 2010a,b; Saavedra et al., 2012). Plant genomes other than that of *A. thaliana* contain only the B subfamily and a small number of genes for PIPK orthologs exist in *C. reinhardtii* (single copy; Awasthi et al., 2012) and *P. patens* (two copies; Saavedra et al., 2009, 2011, 2012). Because red and brown seaweeds as well as green microalgae have only a single copy of the PIPK gene, amplification and functional diversification of PIPK genes also likely occurred during evolution, as in PI-PLC.

As shown in Figure 1, the MORN motifs are not found in PIPKs from red algae, diatoms, and brown *E. siliculosus*, indicating the presence of the MORN repeats is restricted into the green lineage, although PIPKs without the MORN motifs are conserved in *A. thaliana*, as mentioned above. Because the common origin of the red and green algae can be ascribed to a single symbiosis (Keeling, 2010), there are two possibilities for the presence of the MORN motifs in the green lineage. One is that although an ancestral plant cell has a MORN motif-containing PIPK, this motif disappeared from red algae after the divergence of red and green algae. The other is that an ancestral plant cell has a PIPK consisting of the dimerization and catalytic domains and the MORN motifs appeared only in green algae after the divergence of red and green algae. The absence of the MORN motifs in the brown seaweed can be explained by either possibility. According to these findings, the activation mode of PIPK differs between green plants and red and brown algae. Elucidation of the functions of the MORN motif is necessary to fully explain such a difference.

## Conclusions

Comparative genomics provide an evolutionary insight into the taxonomic origin of enzymes and their isoforms, which is generally drawn based on the presence and number of gene family members. Here, it is demonstrated that analysis of domain structure can allow novel evolutionary conclusions. Because domain structure is involved in the regulation of the active state of each protein, comparison of plant genomes with a focus on the structure of and changes in protein domains could open new scenarios regarding the origin and evolution of signaling networks that regulate development and stress responses in plants.

### Conflict of interest statement

The author declares that the research was conducted in the absence of any commercial or financial relationships that could be construed as a potential conflict of interest.
